# Synthesis of (3*S*,3′*S*)- and *meso*-Stereoisomers of Alloxanthin and Determination of Absolute Configuration of Alloxanthin Isolated from Aquatic Animals

**DOI:** 10.3390/md12052623

**Published:** 2014-05-08

**Authors:** Yumiko Yamano, Takashi Maoka, Akimori Wada

**Affiliations:** 1 Kobe Pharmaceutical University, Motoyamakita-machi, Higashinada-ku, Kobe 658-8558, Japan; E-Mail: a-wada@kobepharma-u.ac.jp; 2 Research Institute for Production Development, 15 Shimogamo-morimoto-cho, Sakyo-ku, Kyoto 606-0805, Japan; E-Mail: maoka@mbox.kyoto-inet.or.jp

**Keywords:** carotenoid, alloxanthin, synthesis, chiral HPLC separation, absolute configuration

## Abstract

In order to determine the absolute configuration of naturally occurring alloxanthin, a HPLC analytical method for three stereoisomers **1a**–**c** was established by using a chiral column. Two authentic samples, (3*S*,3′*S*)- and *meso*-stereoisomers **1b** and **1c**, were chemically synthesized according to the method previously developed for (3*R*,3′*R*)-alloxanthin (**1a**). Application of this method to various alloxanthin specimens of aquatic animals demonstrated that those isolated from shellfishes, tunicates, and crucian carp are identical with (3*R*,3′*R*)-stereoisomer **1a**, and unexpectedly those from lake shrimp, catfish, biwa goby, and biwa trout are mixtures of three stereoisomers of **1a**–**c**.

## 1. Introduction

**Figure 1:**
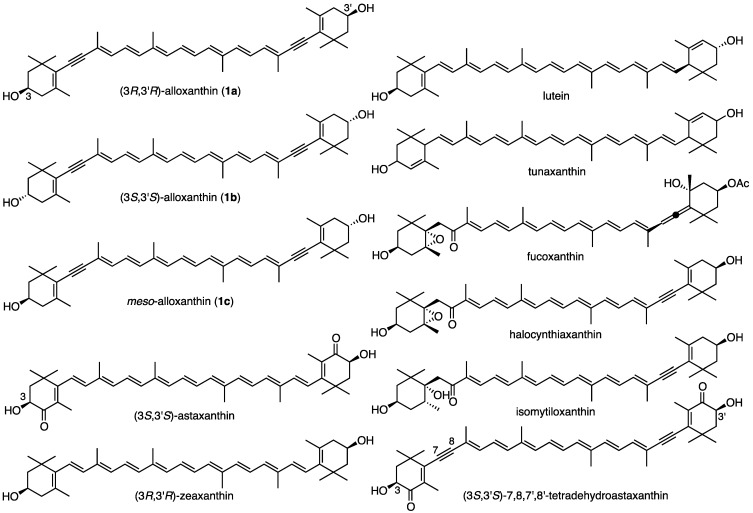
Structures of stereoisomers of alloxanthin (**1a**–**c**) and other related carotenoids.

Alloxanthin (**1**) (Figure 1) was first isolated from *Cryptomonas* algae [1] and its structure was determined to be 7,8,7′,8′-tetreradehydro-β,β-carotene-3,3′-diol by MS, IR and ^1^H-NMR spectroscopies [2]. Additionally, cynthiaxanthin [3] from the tunicate *Cynthia rorezi* (*Halocynthia rorezi*) and pectenoxanthin [4] from giant scallop *Pecten maximus* were isolated by Japanese scientists. In 1967, Campbel *et al.* demonstrated that these two carotenoids were identical with alloxanthin [5]. Therefore, cynthiaxanthin and pectenoxanthin were synonyms of alloxanthin. The absolute configuration of alloxanthin isolated form algae was deduced to be 3*R*,3′*R* by X-ray analysis of degradation product of fucoxanthin and in view of biogenetic grounds [6]. Bartlett *et al.* reported that the ORD spectra of alloxanthin specimens from *Cryptomonas* algae and tunicate showed an identical shape each other and that both specimens are assumed to have an identical absolute configuration [7].

Since then, alloxanthin was isolated from several aquatic animals, such as shellfishes [8,9], starfishes [10], tunicates [11,12] and freshwater fishes [13,14], *etc.* These alloxanthin specimens showed similar non-conservative CD with weak negative Cotton effects. 

Carotenoids such as astaxanthin, zeaxanthin, lutein, and tunaxanthin in animals are known to exist as a mixture of stereoisomers. Namely, astaxanthin in crustaceans and marine fishes exists as a mixture of three stereoisomers at C3 and C3′-positions [15,16]. Zeaxanthin [17], lutein [18], and tunaxanthin [19] in marine fishes also consist of these stereoisomers. Their absolute configurations were determined by CD spectra and chiral HPLC analyses. Due to its non-conservative CD, absolute configurations of alloxanthin in several origins could not be determined exactly by CD spectra. 

In order to determine the absolute configuration of naturally occurring alloxanthin, we synthesized stereoisomers of alloxanthin (**1a**–**c**) and established a HPLC analytical method using a chiral column. Applying this method, the absolute configurations of alloxanthin specimens isolated from shellfishes, tunicates and fishes were investigated. Here, we describe these results. 

## 2. Results and Discussion

### 2.1. Synthesis of (3S,3′S)-Alloxanthin (**1b**) and meso-Alloxanthin (**1c**)

**Scheme 1:**
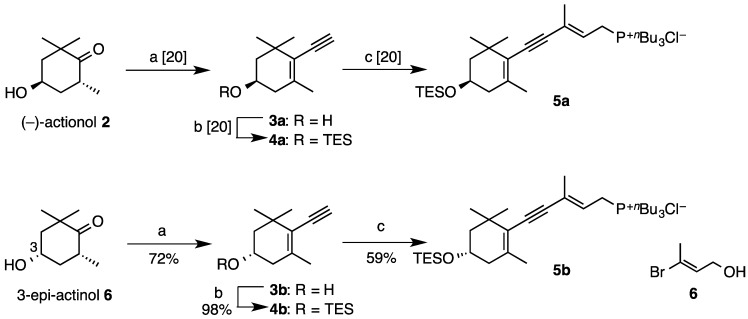
Synthesis of C_15_-acetylenic tri-*n*-butylphosphonium salts **5a** and **5b**.

We previously reported [20] stereoselective total synthesis of (3*R*,3′*R*)-alloxanthin (**1a**) by use of C_15_-acetylenic tri-*n*-butylphosphonium salt **5a** (Scheme 1) as a versatile synthon for syntheses of acetylenic carotenoids. This time, (3*S*,3′*S*)-alloxanthin (**1b**) and its *meso*-stereoisomer **1c** were newly synthesized using (3*S*)-phosphonium salt **5b**, which was prepared from 3-epi-actinol **6** [21] in the same procedure [20] as preparation of (3*R*)-one **5a**. 

Reagents: (**a**) (i) TMSCl, Et_3_N, DMAP, (ii) TMSC≡CH, *^n^*BuLi, then aq. KOH, (iii) Ac_2_O, pyridine, (iv) CuSO_4_, xylene, reflux (Dean-Stark), (v) LiAlH_4_; (**b**) TESCl, Et_3_N, DMAP, (**c**) (i) vinyl bromide **6**, Pd(PPh_3_)_4_, CuI, BHT, *^i^*Pr_2_NH, (ii) MsCl, LiCl, γ-collidine, (iii) P*^n^*Bu_3_, Et_3_N.

Compound **6** was converted into terminal alkyne **3b** via the addition of lithium acetylide in 72% yield over six steps. The high enantiomeric purity of **3b** (99% ee) was confirmed by HPLC analysis [CHIRALPAK AY-H; Daicel, 2-PrOH–*n*-hexane (5:95)]. Compound **3b** was then transformed into the phoshonium salt **5b** via Sonogashira cross-coupling of the triethylsilyl (TES)-protected terminal alkyne **4b** with vinylbromide **6** in 59% over four steps. 

**Scheme 2:**
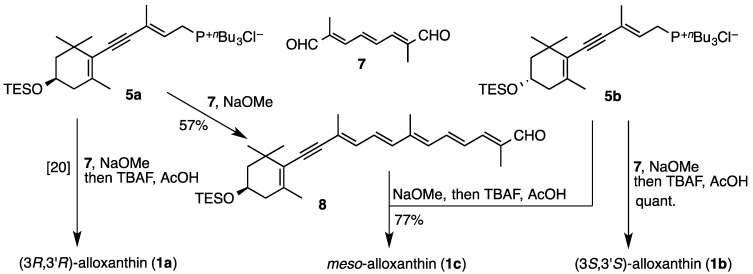
Synthesis of three stereoisomers of alloxanthin (**1a**–**c**).

Wittig condensation of C_10_-dialdehyde **7** with excess amount of (3*S*)-phosphonium salt **5b** in the presence of sodium methoxide in dichloromethane at room temperature and subsequent desilylation stereoselectively provided (3*S*,3′*S*)-alloxanthin (**1b**) (Scheme 2). On the other hand, *meso*-alloxanthin (**1c**) was synthesized via condensation between (3*S*)-phosphonium salt **5b** and (3*R*)-C_25_-acetylenic apocarotenal **8**, which was prepared by Wittig reaction of C_10_-dialdehyde **7** with (3*R*)-phosphonium salt **5a** in the presence of sodium methoxide in dichloromethane at 0 °C. 

**Figure 2:**
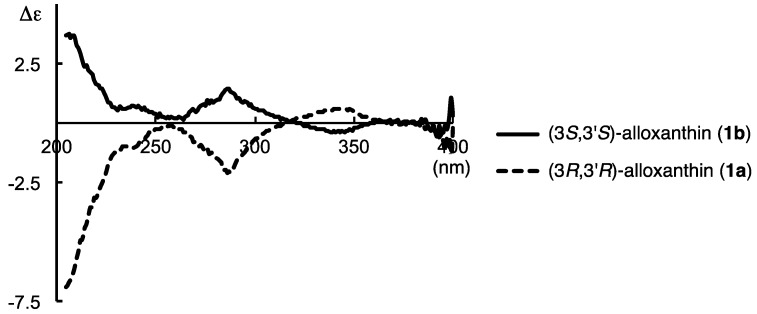
CD spectra in Et_2_O–isopentane–EtOH (5:5:2) of synthesized (3*R*,3′*R*)-alloxanthin (**1a**) and (3*S*,3′*S*)-alloxanthin (**1b**).

CD spectrum of (3*S*,3′*S*)-alloxanthin (**1b**) showed an antisymetrical curve having week Cotton effects to that of previously synthesized [20] (3*R*,3′*R*)-alloxanthin (**1a**) as shown in Figure 2.

### 2.2. Determination of Absolute Configuration of Alloxanthin Isolated from Aquatic Animals by HPLC

**Figure 3:**
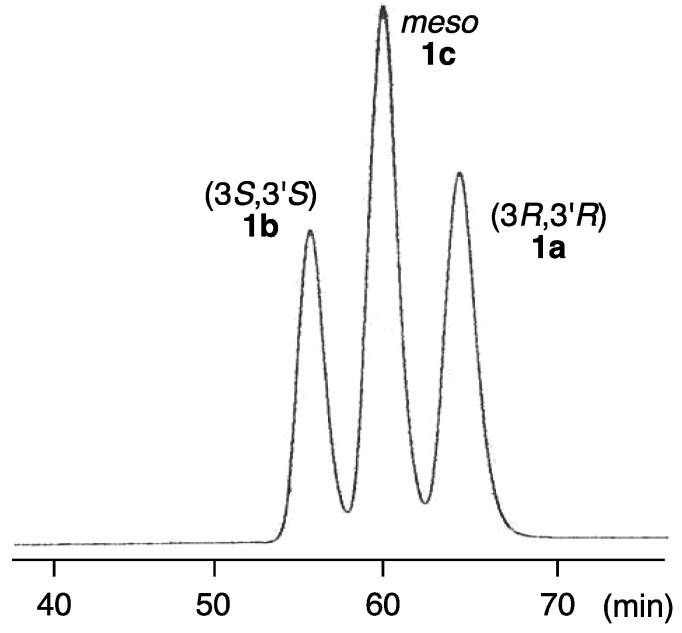
HPLC elution profile of a mixture of three stereoisomers of alloxanthin (**1a**–**c**).

In order to determine the absolute configuration of naturally occurring alloxanthin, a HPLC analytical method for three stereoisomers **1a**–**c** was investigated. As a result, three synthetic stereoisomers of alloxanthin can be separated using a chiral column (CHIRALPAK AD-H; Daicel) as shown in Figure 3.

Next, alloxanthin specimens isolated from scallop *Mizuhopecten yessoensis*, oyster *Crassostrea gigas*, pacific pearl oyster *Pinctada margaritifera*, freshwater bivalve *Unio douglasiae*, tunicate *Halocynthia roretzi*, and crucian carp *Carassius auratus grandoculis* were subjected to the HPLC method to find that these consist of only (3*R*,3′*R*)-stereoisomer **1a**. On the other hand, alloxanthin specimens isolated from lake shrimp *Palaemon paucidens*, catfish *Silurus asotus*, biwa goby *Gymnogobius isaza*, and biwa trout *Oncorhynchus masou rhodurus* consisted of three stereoisomers **1a**–**c** (Table 1).

Column: CHIRALPAK AD-H 0.46 × 25 cm (Daicel, Tokyo, Japan); eluent: 2-PrOH–*n*-hexane (4:96); flow rate: 0.6 mL/min; temperature: 23 °C; detection: 450 nm.

**Table 1:** Occurrence and percentage composition of alloxanthin stereoisomers in aquatic animals.

	Species	3*R*,3′*R*	3*S*,3′*S*	*meso*
		1a	1b	1c
Shellfish				
Scallop	*Mizuhopecten yessoensis*	100	n.d.	n.d.
Oyster	*Crassostrea gigas*	100	n.d.	n.d.
Pacific pearl oyster	*Pinctada margaritifera*	100	n.d.	n.d.
Freshwater bivalves	*Unio douglasiae*	100	n.d.	n.d.
Tunicate				
Sea squirt	*Halocynthia roretzi*	100	n.d.	n.d.
Crustacean				
Lake shrimp	*Palaemon paucidens*	53.7	9.6	36.7
Fish				
Crucian carp	*Carassius auratus grandoculis*	100	n.d.	n.d.
Biwa goby	*Gymnogobius isaza*	91.4	0.9	7.7
Biwa trout	*Oncorhynchus masou rhodurus*	>99.9	trace	trace
Catfish	*Silurus asotus*	82.9	1.5	15.6

n.d.: not detected.

Previously, one of the authors reported that zeaxanthin in plants, shellfishes, and tunicates consisted of only (3*R*,3′*R*)-stereoisomer, whereas zeaxanthin in fishes consisted of three stereoisomers [17]. Similar results were obtained in the case of alloxanthin in aquatic animals. Alloxanthin is *de novo* synthesized in *Chryptophyceae* and *Euglenophyceae* micro algae [22]. However, origin of alloxanthin in aquatic animals was remained uncertain. Patrali *et al.* (1989) [22] and Liaaen-Jensen (1998) [23] reported that alloxanthin in *Mytilus edulis* might be a terminal metabolite of fucoxanthin through intermediates, halocynthiaxanthin and isomytiloxanthin, based on observation in feeding experiment. However, conversion of isomytiloxanthin into alloxanthin is too complex and there were no direct evidences for the conversion, especially in aquatic animals. In our experience, isomytiloxanthin has not been isolated from these animals [24]. 

Shellfishes (bivalves) and tunicates are filter-feeders, which accumulate carotenoids from micro algae. Therefore, alloxanthin in these animals is assumed to originate from *Chryptophyceae* and *Euglenophyceae* micro algae, *etc.* Thus, these alloxanthin specimes consist of only (3*R*,3′*R*)-stereoisomer. Crucian carp is omnivorous and feeds not only animal planktons belonging to Cladocera but also micro algae. Therefore, alloxanthin in crucian carp is also assumed to originate from micro algae. On the other hand, alloxanthin in lake shrimp, catfish, biwa goby, and biwa trout exist as a mixture of three stereoisomres. These crustacean and fishes are carnivorous. Especially, lake shrimp contains a large amount of (3*S*,3′*S*)- and *meso*-alloxanthin (Table 1). Lake shrimp is a one of the major food of catfish and biwa trout. Therefore, (3*S*,3′*S*)- and *meso*-alloxanthin in these fishes might be originated from lake shrimp. However, origin of (3*S*,3′*S*)- and *meso*-alloxanthin in lake shrimp is uncertain. 

Catfish is a top predator in Japanese freshwater ecosystems. Catfish ingests astaxanthin from crustaceans whose astaxanthin exists as a mixture of three stereoisomers. Catfish can convert astaxanthin into zeaxanthin [24]. Therefore, zeaxanthin in catfish exists as a mixture of three stereoisomers. Although the origin of stereoisomers of alloxanthin in catfish is uncertain, it might be naturally formed by epimerization of 7,8,7′,8′-tetradehydroastaxanthin originated from crustacean at C3 and C3′-positions and subsequent reduction at C4 and C4′-positions. Further studies are need to reveal the origin of (3*S*,3′*S*)- and *meso*-alloxanthin in crustaceans and fishes.

This is the first report of the occurrence of (3*S*,3′*S*) and *meso*-alloxanthin in nature.

## 3. Experimental Section

### 3.1. General

IR spectrum was measured on a Perkin-Elmer FT-IR spectrometer (Perkin-Elmer, Yokohama, Japan), spectrum 100. ^1^H and ^13^C NMR spectra were determined on a Varian Gemini-300 superconducting FT-NMR spectrometer (Agilent Technologies, Santa Clara, CA, USA) and the chemical shifts were referenced to tetramethylsilane. Mass spectrum was taken on a Thermo Fisher Scientific Exactive spectrometer (Thermo Fisher Scientific, Bremen, Germany). CD spectra were measured on a Shimadzu-AVIN 62A DS circular dichroism spectrometer (Shimadzu, Kyoto, Japan). 

HPLC analyses were performed on Simadzu-LC-20AT instrument (Shimadzu, Kyoto, Japan) with a photodiode array detector (Waters 996, Tokyo, Japan) and column oven (GL Sciences Model 552, Tokyo, Japan).

NMR assignments are given using the carotenoid nmbering system.

### 3.2. Synthesis of (3S,3′S)-Alloxanthin (**1b**) and meso-Alloxanthin (**1c**)

In the same procedure [20] as preparation of (3*R*)-phosphonium salt **5a** and (3*R*,3′*R*)-alloxanthin (**1a**), (3*S*)-**5b** and (3*S*,3′*S*)-alloxanthin (**1b**) were prepared. Spectral data except for optical data of compounds **1b**, **3b**, **4b** and **5b** were identical with the corresponding previous reported [20] enantiomers **1a**, **3a**, **4a** and **5a**. 

(3*S*,3′*S*)-Alloxanthin (**1b**): HRMS (ESI) *m/z* calcd for C_40_H_53_O_2_ [M + H]^+^ 565.4040, found 565.4038.

Compound **3b**: [α]_D_^26^ 102.9 (*c* 1.03, MeOH); HRMS (ESI) *m/z* calcd for C_11_H_17_O [M + H]^+^ 165.1274, found 165.1277.

Compound **4b**: [α]_D_^23^ 68.1 (*c* 1.00, MeOH); HRMS (ESI) *m/z* calcd for C_17_H_31_OSi [M + H]^+^ 279.2139, found 279.2139.

Compound **5b**: HRMS (ESI) *m/z* calcd for C_33_H_62_OPSi [M − Cl]^+^ 533.4302, found 533.4293.

*meso*-Alloxanthin (**1c**) was synthesized via condensation between **5b** and (3*R*)-C_25_-acetylenic apocarotenal **8**, which was prepared by Wittig reaction of C_10_-dialdehyde **7** with **5a** as follows.

(2*E*,4*E*,6*E*,8*E*,10*E*)-2,7,11-trimethyl-13-[(*R*)-2,6,6-trimethyl-4-triethylsilyloxycyclohex-1-en-1-yl]trideca-2,4,6,8,10-pentaen-12-ynal (**8**). NaOMe (1 M in MeOH; 1.2 mL, 1.2 mmol) was added to a solution of the (3*R*)-phosphonium salt **5a** (409 mg, 0.73 mmol) and C_10_-dialdehyde **7** (100 mg, 0.61 mmol) in CH_2_Cl_2_ (10 mL) at 0 °C. After being stirred at 0 °C for 15 min, the mixture was poured into saturated aq. NH_4_Cl and extracted with AcOEt. The extracts were washed with brine, dried over Na_2_SO_4_ and evaporated to afford a residue, which was purified by flash column chromatography (AcOEt–*n*-hexane, 1:4) to give the (3*R*)-C_25_-acetylenic apocarotenal **8** (165 mg, 57%) as an orange viscous oil: UV-VIS λ_max_ (EtOH)/nm 420; IR ν_max_ (CHCl_3_)/cm^−1^ 2170 (C≡C), 1663 (conj. CHO), 1610 and 1599 (split) (C=C), 1552 (C=C); ^1^H-NMR (CDCl_3_, 300 MHz) δ 0.61 (6H, q, *J* = 8 Hz, SiCH_2_ × 3), 0.97 (9H, t, *J* = 8 Hz, CH_2_CH_3_ × 3), 1.14 and 1.18 (each 3H, s, 1-gem-Me), 1.49 (1H, t, *J* = 12 Hz, 2-H_β_), 1.74 (1H, ddd, *J* = 12, 3.5, 2 Hz, 2-H_α_), 1.89 (3H), 1.91 (3H) and 2.03 (6H) (each s, 5-Me, 9-Me, 13-Me and 13′-Me), 2.11 (1H, br dd, *J* = 17.5, 9.5 Hz, 4-H_β_), 2.30 (1H, br dd, *J* = 17.5, 5.5 Hz, 4-H_α_), 3.94 (1H, m, 3-H), 6.32 (1H, br d, *J* = 12 Hz, 14-H), 6.37 (1H, d, *J* = 15 Hz, 12-H), 6.46 (1H, br d, *J* = 11.5 Hz, 10-H), 6.66 (1H, dd, *J* = 15, 11.5 Hz, 11-H), 6.70 (1H, dd, *J* = 14.5, 11.5 Hz, 15′-H), 6.96 (1H, br d, *J* = 11.5 Hz, 14′-H), 7.03 (1H, dd, *J* = 14.5, 12 Hz, 15-H), 9.46 (1H, s, CHO); ^13^C-NMR (CDCl_3_, 75 MHz) δ 4.82 (C × 3), 6.83 (C × 3), 9.59, 12.96, 18.17, 22.53, 28.61, 30.45, 36.53, 42.11, 47.04, 65.01, 90.10, 98.16, 121.15, 123.84, 126.60, 127.73, 131.75, 134.51, 137.02, 137.07, 137.47, 138.70, 141.26, 148.75, 194.45; HRMS (ESI) *m/z* calcd for C_31_H_47_O_2_Si (MH)^+^ 479.3340, found 479.3347.

Preparation of *meso*-alloxanthin (**1c**). NaOMe (1 M in MeOH; 0.24 mL, 0.24 mmol) was added to a solution of the (3*S*)-phosphonium salt 5b (113 mg, 0.20 mmol) and (3*R*)-C_25_-acetylenic apocarotenal 8 (59 mg, 0.12 mmol) in CH_2_Cl_2_ (10 mL) at room temperature. After being stirred for further 15 min, the mixture was poured into saturated aq. NH_4_Cl and extracted with AcOEt. The extracts were washed with brine, dried over Na_2_SO_4_ and evaporated to afford a residue, which was purified by flash column chromatography (AcOEt–*n*-hexane, 1:4) to give the TES-protected condensed product. Subsequently, to a solution of this condensed product in dry THF (5 mL) were added AcOH (1 M in THF; 0.20 mL, 0.20 mmol) and then tetrabutylammonium fluoride (TBAF) (1 M in THF, 0.40 mL, 0.40 mmol). After being stirred at room temperature for 2 h, the mixture was concentrated to give a residue, which was purified by flash column chromatography (AcOEt–*n*-hexane–MeOH, 50:45:5) to provide *meso*-alloxanthin (**1c**) (70 mg, quant.) as red solids. Its spectral data were identical with those of (3*R*,3′*R*)-alloxanthin (**1a**) [20]. HRMS (ESI) *m/z* calcd for C_40_H_53_O_2_ [M + H]^+^ 565.4040, found 565.4033.

### 3.3. Configurational Analysis of Natural Alloxanthin

#### 3.3.1. Animal Materials

Scallop *Mizuhopecten yessoensis* was provided from Hokkaido Research Organization, Abashiri Fisheries Research Institute, Hokkaido, Japan. Oyster *Crassostrea gigas*, and sea squirt *Halocynthia roretzi* were purchased from fisheries market at Kyoto city. Pacific pearl oyster *Pinctada margaritifera* was provided from a pearl aquaculture industry, Ishigaki city, Okinawa Prefecture. Freshwater bivalve *Unio douglasiae*, crucian carp *Carassius auratus grandoculis*, and catfish *Silurus asotus* were purchased from Katata fisheries cooperative, Shiga Prefecture. Biwa trout *Oncorhynchus masou rhodurus* was purchased from Nango Fisheries Center, Shiga Prefecture. Biwa goby *Gymnogobius isaza* and lake shrimp *Palaemon paucidens* were purchased from fisheries market at Maibara city.

#### 3.3.2. Isolation of Alloxanthin from Aquatic Animals

According to our routine methods, carotenoid was extracted with acetone from animal tissue. The extract was partitioned between Et_2_O–*n*-hexane (1:1) and water in separating funnel. The organic phase was evaporated and saponified with 5% KOH/MeOH at room temperature for 2 h. Then, unsaponifiable compounds were extracted with Et_2_O–*n*-hexane (1:1, v/v) from the reaction mixture by addition of water. The organic layer was dried over Na_2_SO_4_ and evaporated. The residue was subjected to silica gel column chromatography increasing percentage of Et_2_O in *n*-hexane. The fraction eluted with Et_2_O was subjected to HPLC on silica gel with acetone–*n*-hexane (3:7) to afford alloxanthin. Purity of alloxanthin was checked by UV-Vis, ^1^H-NMR, and MS spectral data. Then alloxanthin obtained from aquatic animals was subject to configurational analysis using a chiral column described above.

## 4. Conclusions

In conclusion, we synthesized stereoisomers of alloxanthin (**1a**–**c**) and established a HPLC analytical method using a chiral column to identify them for naturally occurring alloxanthin. Application of this method to various alloxanthin specimens of aquatic animals demonstrated that those isolated from shellfishes, tunicates, and crucian carp are identical with (3*R*,3′*R*)-stereoisomer **1a**, and unexpectedly those from lake shrimp, catfish, biwa goby, and biwa trout are mixtures of three stereoisomers of **1a**–**c**. This is the first report of the occurrence of (3*S*,3′*S*) and *meso*-alloxanthin in nature. The analytical method can be a powerful tool to identify stereoisomers of alloxanthin in nature in a straightforward manner.
